# Phytochemical Profile and Biological Activities of the Roots of *Caesalpinia bonduc* and the Aerial Part of *Heliotropium indicum*

**DOI:** 10.1155/bri/9058679

**Published:** 2026-05-28

**Authors:** Funkè F. Assouma, Durand Dah-Nouvlessounon, Atchadé Pascal Tchogou, Machioud Maxime Sangaré, Gautier Roko, Bawa Boya, Yao Paul Attien, Olaréwadjou Amogou, Rachidatou Kamirou, Basile Konmy, Adolphe Adjanohoun, Lamine Baba-Moussa, Haziz Sina

**Affiliations:** ^1^ Department of Biochemistry and Cell Biology, University of Abomey-Calavi, Abomey-Calavi, Benin, uac.bj; ^2^ National School of Applied Biosciences and Biotechnology of Dassa-Zoumé (ENSBBA), National University of Sciences, Technologies, Engineering, and Mathematics (UNSTIM), Dassa-Zoumé, Benin; ^3^ Department of Animal Physiology, University of Abomey-Calavi, Abomey-Calavi, Benin, uac.bj; ^4^ Department of Biochemistry-Microbiology, University Jean Lorougnon Guédé-Daloa, Daloa, Ivory Coast; ^5^ Southern Agricultural Research Center, National Agronomic Research Institute of Benin, Cotonou, Benin

**Keywords:** analgesic activity, antibiofilm activity, antibacterial activity, Benin, diuretic activity, medicinal plants, phytochemical screening

## Abstract

This study investigates the phytochemical composition and biological activities of *Heliotropium indicum* and *Caesalpinia bonduc*, two medicinal plants widely used in traditional medicine in Benin. In ethnomedicinal practice, *H. indicum* is commonly prepared as decoctions or infusions of the aerial parts and administered orally or topically to treat inflammatory conditions, wounds, fever, and infections, whereas *C. bonduc* roots are typically used in decoction for the management of urinary tract infections, pain, diabetes, and reproductive disorders. The present research aims to generate preliminary scientific data supporting these traditional applications. Aqueous and hydroethanolic (70% ethanol) extracts were prepared from the aerial parts of *H. indicum* and the roots of *C. bonduc*. Preliminary phytochemical screening was performed using standard qualitative colorimetric and precipitation reactions. The antimicrobial activities were evaluated against multiresistant uropathogenic strains using the agar disk diffusion method and broth microdilution for determination of minimum inhibitory concentrations (MICs). Antibiofilm activity was assessed using a crystal violet colorimetric assay. Analgesic and diuretic activities were evaluated in vivo in Wistar rats using hydroethanolic extracts. Acute oral toxicity was assessed following a limit test at 2000 mg/kg in rats. Phytochemical analysis revealed the presence of alkaloids, flavonoids, glycosides, saponins, and tannins in both plant species. Among the tested samples, only the hydroethanolic extract of *C. bonduc* exhibited antibacterial activity against the tested uropathogenic strains, with inhibition zones ranging from 14.6 to 16.6 mm and MIC values between 6 and 6.5 mg/mL. The aqueous extract of *C. bonduc* and both extracts of *H. indicum* did not exhibit detectable antibacterial activity under the experimental conditions. However, all extracts demonstrated antibiofilm activity, inhibiting biofilm formation in *Escherichia coli* and *Staphylococcus* spp. strains by 40.52%–71.93%. Hydroethanolic extracts of both plants showed significant analgesic activity (*p* < 0.05) in the acetic acid–induced writhing test and exhibited measurable diuretic effects in rats. In the acute toxicity study, no mortality or clinical signs of toxicity were observed at 2000 mg/kg during the 14‐day observation period. Hematological and biochemical parameters remained within normal ranges, and histopathological examination of the liver and kidneys revealed no treatment‐related alterations. These findings provide preliminary experimental support for some of the traditional uses of *H. indicum* and *C. bonduc* and suggest that hydroethanolic extracts of these plants warrant further investigation for the isolation and characterization of bioactive compounds.

## 1. Introduction

Biodiversity represents a vital and irreplaceable natural resource essential for human survival. It offers a wealth of biological resources and valuable information that contribute to the health, well‐being, and economic prosperity of human societies [1]. Among the most valuable components of biodiversity are plant species, which play a crucial role in supporting human health and improving the overall quality of life [2]. Many plants contain bioactive compounds that have long been used in traditional medicine to treat various ailments. The use of medicinal plants is based on the belief that they harbor natural substances that can promote health and alleviate disease [3].

Plant‐derived remedies are of great significance in developing countries, where they are frequently employed as primary healthcare treatments. Medicinal plants possess a wide range of pharmacological activities, such as antimicrobial, anti‐inflammatory, analgesic, antidiabetic, and antitumor effects [4]. However, although the therapeutic potential of medicinal plants is widely acknowledged, their use often relies primarily on traditional knowledge transmitted across generations rather than on scientifically validated evidence. This situation creates a significant gap in our comprehensive understanding of the biological properties, efficacy, and safety profiles of many medicinal plant species, as existing evidence is often heterogeneous, context‐dependent, or limited to preliminary studies, despite their widespread and longstanding use in traditional healthcare systems [5, 6].

Despite their widespread use and the recognition of their diverse pharmacological activities, many medicinal plants are still harvested from the wild and consumed by local populations without rigorous and standardized scientific evaluation of their efficacy, optimal dosages, or potential toxicity [5]. Although previous studies have reported antimicrobial, anti‐inflammatory, analgesic, and other biological properties, such evidence often remains preliminary, context‐dependent, or insufficiently standardized to ensure consistent therapeutic outcomes. As a result, important concerns persist regarding the safety, reproducibility, and appropriate use of these plant‐based remedies, particularly in the absence of validated dosage guidelines and comprehensive toxicological assessments. Consequently, further systematic and well‐controlled investigations are required to strengthen existing evidence, clarify uncertainties, and generate reliable data that may support the rational and safe use of medicinal plants in healthcare [6].

Among the medicinal plants used in traditional healthcare are *Caesalpinia bonduc* (L.) Roxb and *Heliotropium indicum*, two medicinal species with high therapeutic value that are widely used in Benin for the treatment of various diseases. *C. bonduc* is a spiny shrub belonging to the Caesalpiniaceae family and is native to the Guineo‐Congolian climatic zone [7]. It is distributed across tropical and subtropical regions, where it is mainly found in disturbed environments [8]. The plant is a highly commercialized and valued medicinal plant in Southern Benin, where it is commonly known as *Adjikountin* [5]. All parts of this plant, including the roots, leaves, and seeds, possess therapeutic value and have been used in traditional medicine for centuries [9]. In ethnomedicinal practice, these plant parts are typically prepared as decoctions, infusions, or powders and administered orally or topically depending on the indication. The roots are commonly used in decoction for the management of prostate‐related disorders and urinary conditions, and the leaves are applied or consumed to relieve menstrual pain, memory loss, and sexual weakness, while the seeds are used, often in powdered or decocted form, for the management of diabetes and malaria; however, these uses are primarily based on traditional knowledge and remain insufficiently validated by rigorous experimental studies [7].

*H. indicum* L., belonging to the Boraginaceae family, is a medicinal herb widely distributed in tropical and subtropical regions of Africa, Asia, and the Americas [10]. It commonly grows in disturbed habitats such as roadsides, waste lands, and cultivated fields [11]. In traditional medicine, the aerial parts of the plant are commonly prepared as decoctions, infusions, or fresh poultices and administered either orally or topically. These preparations are used to treat a range of conditions, including skin infections, wounds, fever, inflammation, and gastrointestinal disorders [12]. Several studies have reported that *H. indicum* contains a variety of bioactive compounds such as alkaloids, flavonoids, and phenolic compounds, which are associated with biological activities including antimicrobial, anti‐inflammatory, antioxidant, and wound‐healing effects [13]. Despite its widespread traditional use in many parts of the world, including West Africa, scientific data regarding its pharmacological activities and toxicity remain limited, highlighting the need for further investigation [14].

The limited scientific investigation into the pharmacological properties of *C*. *bonduc* and *H*. *indicum* represents an important knowledge gap. Most studies have primarily focused on documenting their traditional uses, while fewer investigations have rigorously evaluated their biological activities and potential toxicity under controlled experimental conditions. Given their widespread use in the form of decoctions, infusions, and topical preparations for the management of infectious, inflammatory, and metabolic disorders, it is essential to generate experimental data that can help substantiate these ethnomedicinal claims. Therefore, further research is required to better characterize the pharmacological properties and safety of these species. In this context, the present study evaluates the antimicrobial, analgesic, antibiofilm, diuretic, and acute toxicity profiles of these plants, providing preliminary but important information that may contribute to a better understanding of their therapeutic potential.

## 2. Materials and Methods

### 2.1. Materials

#### 2.1.1. Plant Material

In the present study, the roots of *Caesalpinia bonduc* (voucher no. YH 749/HNB) and the aerial parts of *Heliotropium indicum* (voucher no. YH 751/HNB) were collected in the southern region of Benin in October 2022. They were identified at the National Herbarium of the University of Abomey‐Calavi. The harvested plant parts were washed with water and then dried in the shade in a ventilated laboratory room at 25 ± 2°C for 14 days. The dried materials were then pulverized using an electric grinder and stored in airtight containers at room temperature until use.

#### 2.1.2. Animal Material

Young Wistar rats aged 6–8 weeks and weighing 80–120 g, of both sexes, were used for this study. Animals were obtained from the animal facility of the University of Abomey‐Calavi and housed under standard laboratory environmental conditions (22 ± 2°C, 12‐h light/dark cycle, and relative humidity: 50%–60%). They were allowed to acclimatize for 1 week with free access to standard rodent feed and water. All animal experiments were conducted in accordance with the ARRIVE guidelines [15] and the principles of laboratory animal care, and the study protocol was approved by the Doctoral School of Life and Earth Sciences of the University of Abomey‐Calavi (Approval No. UAC/EDSVT/11614210).

All procedures involving animals were carried out in accordance with institutional guidelines and ethical approval. Anesthesia was induced using chloroform (Sigma‐Aldrich, USA) administered by inhalation in a closed chamber containing cotton impregnated with approximately 3 mL of chloroform, allowing controlled vapor exposure. Animals were carefully monitored until loss of the righting reflex and absence of response to external stimuli were confirmed. At the end of the experimental period, animals were euthanized under deep chloroform anesthesia using a higher exposure volume (approximately 5 mL per chamber). Death was confirmed by the absence of respiratory movements and reflexes before sample collection. All efforts were made to minimize animal suffering and to reduce the duration of exposure to the minimum required to achieve adequate anesthesia.

All procedures involving animals were performed in compliance with institutional ethical standards and under veterinary supervision. Anesthesia for all invasive procedures was induced using analytical‐grade chloroform (Sigma‐Aldrich, USA) administered by inhalation in a closed chamber, with animals continuously monitored until loss of righting reflex and absence of response to external stimuli were achieved. At the end of the experimental period, animals were euthanized under deep chloroform anesthesia, and death was confirmed by the absence of respiratory movements and reflexes before sample collection.

We acknowledge that chloroform is no longer recommended as an anesthetic agent under current international guidelines due to animal welfare considerations. However, its use in the present study was formally approved by the institutional ethics committee and reflects established local laboratory practices at the time the experiments were conducted. All procedures were carried out under controlled conditions to ensure rapid induction of deep anesthesia and to minimize animal distress, and no anesthesia‐related adverse effects were observed. In line with current recommendations, future studies will preferentially employ refined anesthetic agents with improved safety profiles.

#### 2.1.3. Microbial Strains

Clinical strains of *Staphylococcus* spp. and *Escherichia coli* used in this study were obtained from previously characterized collections reported by Assouma et al. [16, 17], in which their antimicrobial resistance profiles and phenotypic characteristics were established. These isolates were used with appropriate authorization.

### 2.2. Phytochemical Screening

Preliminary phytochemical screening was performed on the powdered plant materials using standard qualitative methods described by Houghton and Raman [18]. Tests were carried out to detect the presence of major secondary metabolite classes, including alkaloids, flavonoids, tannins, saponins, terpenoids, steroids, glycosides, and phenolic compounds, based on characteristic color reactions and precipitation tests.

### 2.3. Sample Preparation and Extraction

#### 2.3.1. Aqueous Extraction

Aqueous extracts were prepared by maceration, a commonly used method in ethnopharmacological studies [19]. Fifty grams (50 g) of plant powder was macerated in 500 mL of distilled water (1:10 w/v) under continuous magnetic stirring at room temperature (25 ± 2°C) for 72 h. To ensure aseptic conditions, all glassware and containers were sterilized by autoclaving at 121°C for 15 min before use. The plant powder was not subjected to sterilization to avoid potential alteration or degradation of thermolabile phytoconstituents; however, it was handled under aseptic conditions and stored in sterile, airtight containers before use. The mixture was filtered twice through sterile cotton wool and once through Whatman No. 1 filter paper. The filtrate was dried in a ventilated oven at 50°C until constant weight.

#### 2.3.2. Hydroethanolic Extraction

Hydroethanolic extracts were prepared using 70% ethanol (v/v) under the same maceration conditions (50‐g powder in 500‐mL solvent for 72 h). The use of ethanol (70% v/v) inherently limits microbial growth during extraction, further contributing to aseptic processing conditions. After filtration, the extract was concentrated under reduced pressure using a rotary evaporator at 50°C, followed by oven drying at 50°C until constant weight. All dried extracts were weighed and stored in sterile containers at 4°C until use.

### 2.4. Evaluation of Antimicrobial Activity

#### 2.4.1. Disk Diffusion Assay

Antibacterial activity of aqueous and hydroethanolic extracts was evaluated using the Kirby–Bauer disk diffusion method, a standardized technique widely used for antimicrobial susceptibility testing [20, 21]. In the present study, this method was used as a preliminary qualitative screening tool to rapidly identify extracts exhibiting antibacterial activity before quantitative determination of minimum inhibitory concentration (MIC) values. Extracts were tested at 20 mg/mL. Sterile 6‐mm filter paper discs (Whatman No. 1) were impregnated with 20 μL of extract solution and placed on Mueller–Hinton agar plates (Biomark Laboratories, India) previously inoculated with bacterial suspensions.

Bacterial inocula were prepared from fresh overnight cultures and adjusted to the 0.5 McFarland turbidity standard using sterile saline solution (0.85% NaCl, Sigma‐Aldrich, USA), corresponding to approximately 1 × 10^8^ CFU/mL. Dimethyl sulfoxide (DMSO, analytical grade, Sigma‐Aldrich, USA) at 5% (v/v) was used to dissolve the extracts, which served as the negative control. The plates were incubated at 37°C for 24 h, after which the diameter of inhibition zones around each disc was measured in millimeters using a calibrated ruler. The size of the inhibition zone was used as an indicator of the antibacterial activity of the tested extracts [21].

#### 2.4.2. Determination of MIC and Minimum Bactericidal Concentration (MBC)

MIC was determined using the broth macrodilution method according to standard antimicrobial susceptibility testing procedures [21, 22]. Serial two‐fold dilutions of the extracts were prepared in Mueller–Hinton broth (Biomark Laboratories, India) to obtain concentrations ranging from 0.156 to 20 mg/mL.

Bacterial suspensions were prepared from 18 to 24‐h cultures and diluted in sterile broth to obtain a final inoculum concentration of approximately 1 × 10^6^ CFU/mL. Each test tube contained a final volume of 2 mL, consisting of 1 mL of extract dilution and 1 mL of bacterial inoculum. Growth control tubes (containing inoculated broth without extract) and sterility control tubes (broth only) were included.

All tubes were incubated at 37°C for 24 h. The MIC was defined as the lowest concentration of extract that completely inhibited visible bacterial growth after incubation. To determine the MBC, aliquots (100 μL) from tubes showing no visible growth were subcultured onto Mueller–Hinton agar plates and incubated at 37°C for 24 h. The MBC was defined as the lowest concentration that produced no bacterial colony growth on agar plates [22]. The antibacterial effect of the extracts was interpreted based on the MBC/MIC ratio: an extract was considered bactericidal when MBC/MIC ≤ 4 and bacteriostatic when MBC/MIC > 4 [23].

### 2.5. Evaluation of the Antibiofilm Activity

Antibiofilm activity was assessed using the crystal violet microtiter plate assay, a widely used method for quantifying bacterial biofilm formation [24, 25]. All reagents used were of analytical grade (Sigma‐Aldrich, USA), and assays were performed under aseptic conditions. Briefly, a bacterial suspension adjusted to approximately 10^8^ CFU/mL was added to each well of a sterile 96‐well microplate, followed by the addition of 100 μL of plant extract solution at a concentration of 20 mg/mL.

Although the extracts of *Heliotropium indicum* did not exhibit antibacterial activity in preliminary assays, they were included in the biofilm experiments to evaluate potential antibiofilm effects independent of planktonic growth inhibition. This approach is supported by previous studies showing that certain plant‐derived compounds can inhibit biofilm formation by interfering with bacterial adhesion, quorum sensing, or extracellular polymeric substance production without affecting bacterial viability [26, 27].

The microplates were incubated at 37°C for 48 h to allow biofilm formation. After incubation, nonadherent cells were carefully removed by washing the wells with sterile distilled water. The plates were then air‐dried for 30 min, and the adhered biofilms were stained with 200 μL of 0.1% (w/v) crystal violet solution for 15 min. Excess stain was removed by washing the wells three times with sterile distilled water. The bound dye was subsequently solubilized using 95% ethanol, and the optical density was measured at 570 nm using a microplate reader (BioTek Instruments, USA). The percentage of biofilm inhibition was calculated according to the following formula:

(1) PIB%=ODcontrol−ODtestODcontrol×100.

### 2.6. Evaluation of the Analgesic Activity

Analgesic activity was assessed using the acetic acid–induced writhing test, a widely used experimental model for evaluating peripheral analgesic activity in rodents [28, 29]. Rats were randomly divided into four experimental groups with six animals in each group. The first group received physiological saline (0.9% NaCl, B. Braun, Germany) and served as the negative control, while the second group received aspirin (100 mg/kg, Sigma‐Aldrich, USA) as the positive control [30]. The third and fourth groups were treated with hydroethanolic extracts of *C. bonduc* or *H. indicum,* respectively, administered orally at a dose of 200 mg/kg body mass.

The experimental design was preventive, as extracts were administered before the induction of nociception. This approach was selected to reflect traditional ethnomedicinal practices in which plant preparations are often used to prevent or attenuate pain symptoms at early stages. Thirty minutes after treatment, abdominal writhing was induced by intraperitoneal injection of 3% acetic acid (10 mL/kg; Sigma‐Aldrich, USA). Following acetic acid administration, the number of abdominal constrictions (writhes) was recorded for 30 min. Analgesic activity was expressed as the percentage inhibition of writhing and calculated according to the following formula:

(2) PIC%=NCC−NCTNCC×100.

where NCC is the mean number of writhes in the control group and NCT is the mean number in the treated group.

### 2.7. Evaluation of the Diuretic Activity

Diuretic activity was assessed using the slight modification method described by Lipschitz et al. [31] and Rahman et al. [32]. Rats were randomly divided into four groups of six animals each. The first group received physiological saline (0.9% NaCl, B. Braun, Germany) and served as the negative control, while the second group received furosemide (10 mg/kg, Sanofi, France) as the positive control. The third and fourth groups were treated orally with hydroethanolic extracts of *C. bonduc* or *H. indicum,* respectively, at a dose of 200 mg/kg body mass. Prior to treatment, animals were hydrated with saline at a dose of 25 mL/kg body mass and placed individually in metabolic cages to allow urine collection. Urine samples were collected over a period of 6 h following administration of the treatments, and the total volume was measured. The concentrations of urinary electrolytes, including Na^+^, K^+^, and Cl^−^, were determined using an electrolyte analyzer (Roche Diagnostics, Switzerland). The volumetric urinary excretion (EUV, %) was calculated using the following formula:

(3) EUV%=volume excretedvolume administered×100.

### 2.8. Evaluation of the Acute Toxicity

Acute oral toxicity of the hydroethanolic extracts of *Caesalpinia bonduc* and *Heliotropium indicum* was evaluated in rats according to the OECD guideline 423 for acute oral toxicity testing [33]. Animals received a single oral limit dose of 2000 mg/kg body mass and were observed for a period of 14 days to assess potential toxic effects. Following extract administration, animals were closely monitored during the first 8 h, with particular attention during the first 30 min, 1 h, 2 h, and 4 h after treatment, and thereafter once daily for the remaining observation period. Parameters evaluated included behavioral changes, locomotor activity, food and water intake, mortality, and variations in body mass. At the end of the 14‐day observation period, blood samples were collected for hematological analysis, including red blood cell count (RBC), white blood cell count (WBC), and hemoglobin (HB) concentration, as well as biochemical analysis of liver and kidney function markers such as ALT, AST, urea, and creatinine. Subsequently, animals were sacrificed, and liver and kidney tissues were collected, fixed in 10% buffered formalin, embedded in paraffin, sectioned, and stained with hematoxylin and eosin for histopathological examination under a light microscope.

### 2.9. Statistical Analysis of Data

All experiments were performed in triplicate for in vitro assays, and results are expressed as mean ± standard deviation (SD). For in vivo experiments, data were obtained from six animals per group (*n* = 6) and are also presented as mean ± SD. Statistical analyses were performed using SPSS Version 26.0 (IBM SPSS, Chicago, IL, USA) and GraphPad Prism Version 9.0.2 (GraphPad Software, USA). The normality of data distribution was assessed using the Shapiro–Wilk test. For normally distributed data, differences between groups were analyzed using one‐way ANOVA followed by Dunnett’s multiple comparison post hoc test to compare treated groups with the control group. For data that did not follow a normal distribution, the Kruskal–Wallis nonparametric test was applied, followed by Dunn’s post hoc test for multiple comparisons. Differences between male and female animals were also evaluated using two‐way ANOVA, considering sex and treatment as independent factors. A *p* value < 0.05 was considered statistically significant.

## 3. Results

### 3.1. Phytochemical Screening

The phytochemical screening of the powdered plant materials revealed the presence of several secondary metabolites associated with promising biological activities (Table 1). Among the identified compounds, we found tannins (gallotannins and catechins), leucoanthocyanins, reducing compounds (defined here as molecules capable of donating electrons in redox reactions, including reducing sugars and certain phenolic compounds detected by standard reduction‐based assays), coumarins, and saponins. However, anthocyanins, cyanogenic derivatives, O‐glycosides, and C‐glycosides were absent in both plants. Alkaloids were detected exclusively in the roots of *Caesalpinia bonduc*, whereas mucilages were observed only in the aerial parts of *Heliotropium indicum*.

**TABLE 1:** Secondary metabolites detected in the roots of *C*. *bonduc* and the aerial parts of *H*. *indicum*.

Secondary metabolites	Roots of *C. bonduc*	Aerial parts of *H. indicum*
Tannin	−	+
Catechic tannins	+	+
Leucoanthocyanins	+	+
Gallotannins	+	+
Flavonoids	−	−
Anthocyanins	−	−
Saponins	+	+
Cyanogenic derivatives	−	−
Mucilages	−	+
Reducing compounds (reducing sugars and phenolic reductants)	+	+
Free anthraquinones	−	−
Alkaloids	+	−
Quinone derivatives	−	−
Coumarins	+	+
O‐glycosides	−	−
C‐glycosides	−	−

*Note:* “+” indicates the presence of the compound; “−” indicates its absence.

### 3.2. Antimicrobial Activity

The antibacterial activity of aqueous and hydroethanolic extracts against uropathogenic strains is presented in Table 2. Only the hydroethanolic extract of *Caesalpinia bonduc* inhibited the proliferation of *Escherichia coli* and *Staphylococcus* spp., with inhibition zone diameters ranging from 14.6 ± 1.14 mm to 16.6 ± 2.41 mm, with statistically significant differences observed at the level of each bacterial group when comparing the different extracts (*p* < 0.05). In contrast, no antibacterial activity was observed with *H*. *indicum* extracts or *C*. *bonduc* aqueous extracts.

**TABLE 2:** Inhibition zone diameter (mm) of the extracts against the tested bacterial strains.

	HIA	HIE	CBA	CBE	*p* value
*Escherichia coli*	00	00	00	14.6 ± 1.14	0.003
*Staphylococcus* spp.	00	00	00	16.6 ± 2.41	0.007

*Note:* Values are expressed as mean ± standard deviation. “00” indicates no detectable inhibition zone. Statistical comparisons were performed between extracts for each bacterial strain.

Abbreviations: CBA = *Caesalpinia bonduc* aqueous, CBE = *C. bonduc* hydroethanolic, HIA = *Heliotro*pium *indicum* aqueous, and HIE = *H. indicum* hydroethanolic.

Consequently, MIC and MBC determinations were performed exclusively for the hydroethanolic extract of *C. bonduc*, while no MIC values were determined for *H. indicum* extracts due to the absence of detectable inhibition in the initial screening assay.

The hydroethanolic extract of *Caesalpinia bonduc* roots exhibited MICs of 6.5 ± 3.35 mg/mL against *Escherichia coli* and 6.0 ± 2.24 mg/mL against *Staphylococcus* spp*.* (Table 3). The corresponding MBC was 18.0 ± 4.47 mg/mL for both bacterial groups. The calculated MBC/MIC ratios were 2.77 for *Escherichia coli* and 3.00 for *Staphylococcus* spp., based on established interpretative criteria reported in antimicrobial susceptibility studies, where a ratio ≤ 4 indicates bactericidal activity. These results therefore indicate that while the hydroethanolic extract exhibits a bactericidal effect against the tested uropathogenic strains, the relatively high MIC values suggest a moderate antibacterial potency.

**TABLE 3:** Minimum inhibitory concentrations (MICs) and minimum bactericidal concentrations (MBCs) of the extracts against bacterial strains.

Parameter	Strain	CBE (mean ± SD)	HIA	HIE	CBA
MIC	*Escherichia coli*	6.50 ± 3.35	‐	‐	‐
	*Staphylococcus* spp.	6.00 ± 2.24^ab^	‐	‐	‐

MBC	*Escherichia coli*	18.00 ± 4.47	‐	‐	‐
	*Staphylococcus* spp.	18.00 ± 4.47	‐	‐	‐

MIC/MBC	*Escherichia coli*	0.35 ± 0.14	‐	‐	‐
	*Staphylococcus* spp.	0.45 ± 0.11^b^	‐	‐	‐

*Note:* Values are expressed as mean ± standard deviation. “‐” indicates no detectable inhibition zone. Statistical comparisons were performed between extracts for each bacterial strain. Means followed by the same letters (a, b) are not significantly different at the 5% significance level (*p* > 0.05).

Abbreviations: CBA = Caesalpinia bonduc aqueous, CBE = C. bonduc hydroethanolic, HIA = *Heliotropium indicum* aqueous, and HIE = *H. indicum* hydroethanolic.

### 3.3. Biofilm Inhibition Activity

The inhibitory effects of the plant extracts on bacterial biofilm formation are presented in Table 4. Although *Heliotropium indicum* extracts did not inhibit bacterial growth in preliminary assays, they were included in the biofilm experiments to assess potential antibiofilm effects independent of antibacterial activity. All tested extracts demonstrated the ability to inhibit biofilm formation on enterobacterial and staphylococcal strains. The percentages of biofilm inhibition ranged from 40.52% to 71.93%, depending on the extract and bacterial group.

**TABLE 4:** Percentage of biofilm inhibition by the extracts.

Extract	*Escherichia coli* (%)	*Staphylococcus* spp. (%)
HIA	53.37 ± 11.43	40.52 ± 7.18
HIE	55.16 ± 17.11	71.93 ± 8.74
CBA	47.91 ± 14.45	44.44 ± 20.54
CBE	45.08 ± 6.77	46.29 ± 11.66
*p* value	0.536	0.004

Abbreviations: CBA = *Caesalpinia bonduc* aqueous, CBE = *C*. *bonduc* hydroethanolic, HIA = *Heliotropium indicum* aqueous, and HIE = *H. indicum* hydroethanolic.

For *Escherichia coli*, inhibition values varied between 45.08% and 55.16%, with no statistically significant difference among the tested extracts (*p* = 0.536). In contrast, significant differences were observed for *Staphylococcus* spp. (*p* = 0.004), with the hydroethanolic extract of *H*. *indicum* showing the highest inhibition rate (71.93 ± 8.74%). These results indicate that both plant species possess compounds capable of interfering with bacterial biofilm formation.

### 3.4. Analgesic Activity

The analgesic effects of the hydroethanolic extracts are presented in Table 5. Both extracts significantly reduced acetic acid–induced writhing in rats compared with the negative control (*p* < 0.05). The percentage inhibition of writhing was 59.32 ± 3.62% for the hydroethanolic extract of *Caesalpinia bonduc* and 64.41 ± 5.44% for the hydroethanolic extract of *Heliotropium indicum*.

**TABLE 5:** Effects of the extracts on acetic acid–induced writhing.

Extract	PIC (%)	Effect vs. NC (*p* value)	Effect vs. PC (*p* value)
CBE	59.32 ± 3.62	0.0021	0.9999
HIE	64.41 ± 5.44	0.0001	0.9999
PC	61.02 ± 5.44	0.0003	
NC	0		0.0003

*Abbreviations:* CBE = *Caesalpinia bonduc* hydroethanolic, HIE = *Heliotropium indicum* hydroethanolic, NC = negative control, and PC: positive control.

No statistically significant difference was observed between the extracts and the positive control (aspirin) (*p* > 0.05), suggesting that the analgesic activity of the plant extracts is comparable to that of the standard analgesic drug under the experimental conditions.

### 3.5. Diuretic Activity

The effects of the hydroethanolic extracts of *Heliotropium indicum* and *Caesalpinia bonduc* on urinary output and electrolyte excretion are presented in Table 6. Administration of the extracts resulted in a significant increase in urinary output compared with the negative control group (*p* < 0.0001). The urinary excretion volume (UEV) reached 74.3 ± 13.2 mL for *Heliotropium indicum* and 85.5 ± 15.7 mL for *Caesalpinia bonduc*, values comparable to those obtained with the reference diuretic drug furosemide. Similarly, both extracts significantly increased the urinary excretion of sodium (Na^+^), potassium (K^+^), and chloride (Cl^−^) ions relative to the control group, indicating enhanced electrolyte elimination.

**TABLE 6:** Effect of the hydroethanolic extracts on urinary volume and electrolyte excretion.

Extracts	UEV (mL)	Na (mEq/L)	K (mEq/L)	Cl (mEq/L)	*p* value
HIE	74.3 ± 13.2	148.1 ± 12.8	64.8 ± 8.7	103.6 ± 8	< 0.0001
CBE	85.5 ± 15.7	158.8 ± 21.2	77.8 ± 5.9	111.1 ± 10	< 0.0001
NC	26.7 ± 7.5	104.3 ± 1.2b	43 ± 0.99b	69.19 ± 1.5b	< 0.0001
PC	88.74 ± 1.0	166.7 ± 4.8	78.7 ± 2.4	119.1 ± 6.8	< 0.0001

*Note:* Values are expressed as mean ± standard deviation (*n* = 6). Within each column, values sharing different superscript letters (a, b) are significantly different at *p* < 0.05 (post hoc Tukey test). CBE = *Caesalpinia bonduc* hydroethanolic; HIE = *Heliotropium indicum* hydroethanolic.

Abbreviations: NC = negative control; PC = positive control.

To further characterize the relationship between diuresis and electrolyte handling, Pearson correlation analyses were performed between UEV and electrolyte concentrations (Na^+^, K^+^, and Cl^−^). The results revealed strong positive correlations between UEV volume and all measured ions. The correlation coefficients between UEV and Na+, K+, and Cl‐ were 0.978, 0.967, and 0.968, respectively (Table 7), indicating a proportional increase in electrolyte excretion with rising urine output.

**TABLE 7:** Correlation matrix between urinary excretion volume and electrolytes.

	**UEV**	**Na**	**K**	**Cl**

UEV	1	0.978	0.967	0.968
Na	0.978	1	0.987	0.99
K	0.967	0.987	1	0.983
Cl	0.968	0.99	0.983	1

*Note:* Values represent Pearson correlation coefficients (*r*).

Similarly, strong positive correlations were observed among the electrolytes themselves (Na^+^–K^+^, *r* = 0.987; Na^+^–Cl^-^, *r* = 0.990; K^+^–Cl^-^, *r* = 0.983), suggesting coordinated renal excretion mechanisms.

### 3.6. Oral Toxicity of the Extracts

No mortality or visible signs of toxicity were observed in rats treated with the hydroethanolic extracts of *Caesalpinia bonduc* and *Heliotropium indicum* during the 14‐day observation period. Animals showed normal behavior, motor coordination, and physiological appearance, including normal characteristics of feces and urine. A significant increase in body mass was observed during the study period (*p* < 0.05), suggesting normal growth and absence of adverse systemic effects (Table 8).

**TABLE 8:** Variation in body mass of rats.

Rats	CBE (g)	HIE (g)	*p* value
Day 0	155.67 ± 16.26	163 ± 15.62	0.096
Day 14	168 ± 19.16	171.67 ± 14.50	0.41
Gain	12.33 ± 3.52ab	8.67 ± 7.23a	0.003

*Note:* CBE = *Caesalpinia bonduc* hydroethanolic; HIE = *Heliotropium indicum* hydroethanolic.

Hematological analysis revealed no significant alterations in most evaluated parameters between Day 0 and Day 14 (Table 9). More specifically, erythrocyte‐related indices, including RBC, HB, and hematocrit (HCT), remained globally stable across the experimental period, although a statistically significant variation in RBC (*p* = 0.03) and HCT (*p* = 0.04) was observed between extracts, with a moderate increase in HCT, particularly in the HIE‐treated group. Similarly, erythrocyte indices such as mean corpuscular volume (MCV) showed a significant increase (*p* = 0.02), suggesting a possible adaptive hematological response rather than a toxic effect, while mean corpuscular hemoglobin (MCH) and mean corpuscular hemoglobin concentration (MCHC) remained unchanged, indicating preserved red cell integrity.

**TABLE 9:** Effects of hydroethanolic extracts on hematological parameters in rats.

Extracts	CBE	HIE	SEM	*p* value
*RBC (×* 10^6^ */μL)*				
Day 0	8.38	8.43	0.29	0.91
Day 14	7.95	8.99	0.36	0.07
Variation	−0.43^a^	0.57^b^	0.20	0.03

*HB (g/dL)*				
Day 0	14.77	14.80	0.47	0.92
Day 14	14.90	16.10	0.27	0.05
Variation	0.13	1.30	0.41	0.08

*HCT (%)*				
Day 0	46.66	45.67	1.06	0.83
Day 14	52.00	57.67	1.80	0.08
Variation	5.33^ab^	12.00^b^	1.17	0.04

*MCV (fL)*				
Day 0	55.33	54.67	1.13	0.69
Day 14	65.67	64.00	1.39	0.10
Variation	10.33^ab^	9.33^a^	0.62	0.02

*MCH (pg)*				
Day 0	17.67	17.33	0.27	0.45
Day 14	18.33	18.00	0.70	0.26
Variation	0.66	0.67	0.64	0.59

*MCHC (g/dL)*				
Day 0	31.33	31.67	0.44	0.93
Day 14	28.00	28.00	0.58	0.73
Variation	−3.33	−3.67	0.86	0.97

WBC (× 10^3^/μL)				
Day 0	8.04	7.27	0.81	0.60
Day 14	7.72	7.39	0.98	0.96
Variation	−0.32	0.11	1.01	0.87

*Neutrophils (%)*				
Day 0	11.00	7.00	5.88	0.96
Day 14	13.33	10.67	4.11	0.57
Variation	2.33	3.67	6.55	0.92

*Eosinophils (%)*				
Day 0	0.66	1.67	0.67	0.75
Day 14	0.66	1.00	0.38	0.47
Variation	0.00	−0.67	0.83	0.42

*Monocytes (%)*				
Day 0	17.33	17.33	5.95	0.81
Day 14	15.66	14.67	3.03	0.87
Variation	−1.66	−2.67	6.36	0.80

*Lymphocytes (%)*				
Day 0	71.00	74.00	2.36	0.01
Day 14	70.33	73.67	1.93	0.14
Variation	−0.67	−0.33	3.12	0.25

*Platelets (×* 10^3^ */μL)*				
Day 0	757.67	758.67	67.75	0.78
Day 14	653.67	720.33	61.24	0.56
Variation	−104.00	−38.33	50.19	0.35

*Note:* HB = hemoglobin, HCT = hematocrit, PLT = platelets, NEUTRO = neutrophils, EOS = eosinophils, MONO = monocytes, and LYMPHO = lymphocytes. CBE = *Caesalpinia bonduc* hydroethanolic, HIE = *Heliotropium indicum* hydroethanolic, and SEM = standard error of the mean. Different letters in the same row indicate statistically significant differences (*p* < 0.05).

Abbreviations: MCH = mean corpuscular hemoglobin, MCHC = mean corpuscular hemoglobin concentration, MCV = mean corpuscular volume, RBC = red blood cells, and WBC = white blood cells.

Leukocyte parameters, including total WBC and differential counts (neutrophils, eosinophils, monocytes, and lymphocytes), did not show significant variations over time or between treatments (*p* > 0.05), reflecting the absence of inflammatory or immunotoxic responses induced by the extracts. Platelet counts also remained statistically stable despite slight decreases, further supporting the hematological safety profile of both extracts under the tested conditions.

Similarly, biochemical parameters related to liver and kidney function showed no clinically relevant variations between treated groups (Table 10). Markers of renal function, including urea and creatinine, remained within normal physiological ranges, although creatinine exhibited a statistically significant variation between groups at Day 14 (*p* = 0.02), with a decrease in the CBE group and a slight increase in the HIE group; however, these changes were not indicative of renal impairment.

**TABLE 10:** Effects of hydroethanolic extracts on biochemical parameters in rats.

Extracts	CBE	HIE	SEM	*p* value
*GLY (g/L)*				
Day 0	0.38	0.46	0.03	0.06
Day 14	0.35	0.43	0.05	0.29
Variation	−0.02	−0.03	0.03	0.70

*UREA (g/L)*				
Day 0	0.56	0.53	0.06	0.46
Day 14	0.52	0.42	0.04	0.47
Variation	−0.04	−0.10	0.04	0.27

*CREAT (mg/L)*				
Day 0	7.95	6.26	0.50	0.15
Day 14	6.22^a^	7.60^b^	0.30	0.02
Variation	−1.72^a^	1.34^b^	0.39	0.03

*TC (g/L)*				
Day 0	1.17	1.17	0.04	0.87
Day 14	1.40	1.38	0.15	0.69
Variation	0.22	0.21	0.14	0.74

*HDL (g/L)*				
Day 0	0.44	0.44	0.04	0.59
Day 14	0.42	0.47	0.04	0.54
Variation	−0.02	0.03	0.04	0.34

*TRY (g/L)*				
Day 0	0.90	0.85	0.08	0.16
Day 14	0.88^ab^	1.00^b^	0.08	0.03
Variation	−0.01	0.14	0.08	0.11

*AST (U/L)*				
Day 0	18.00	22.66	2.52	0.44
Day 14	31.00	27.66	6.98	0.14
Variation	13.00	5.00	5.32	0.07

*ALT (U/L)*				
Day 0	24.33	21.66	3.96	0.65
Day 14	40.66	36.00	3.60	0.13
Variation	16.33	14.33	2.61	0.26

*Note:* CBE = *Caesalpinia bonduc* hydroethanolic; HIE = *Heliotropium indicum* hydroethanolic. Different superscript letters (a, b) indicate significant differences between treatments (*p* < 0.05). GLY = blood glucose, UREA = urea, CREAT = creatinine, HDL = high‐density lipoprotein cholesterol, TRY = triglycerides, AST = aspartate aminotransferase, and ALT = alanine aminotransferase.

Abbreviations: SEM = standard error of the mean; TC = total cholesterol.

Hepatic enzymes (AST and ALT) showed moderate increases over time in both groups, but these variations were not statistically significant (*p* > 0.05) and remained within acceptable physiological limits, suggesting no hepatocellular damage. Lipid profile parameters, including total cholesterol (TC), high‐density lipoprotein (HDL), and triglycerides (TRY), did not exhibit biologically relevant alterations, although TRY showed a statistically significant difference at Day 14 (*p* = 0.03), which was not associated with a consistent pathological trend. Blood glucose levels also remained stable throughout the experimental period.

Overall, despite some statistically significant variations in specific parameters, none of these changes followed a consistent dose‐dependent or pathological pattern. Collectively, these findings support the absence of hematological, renal, or hepatic toxicity associated with the administration of the hydroethanolic extracts under the experimental conditions.

### 3.7. Histopathological Study Results

**FIGURE 1:**
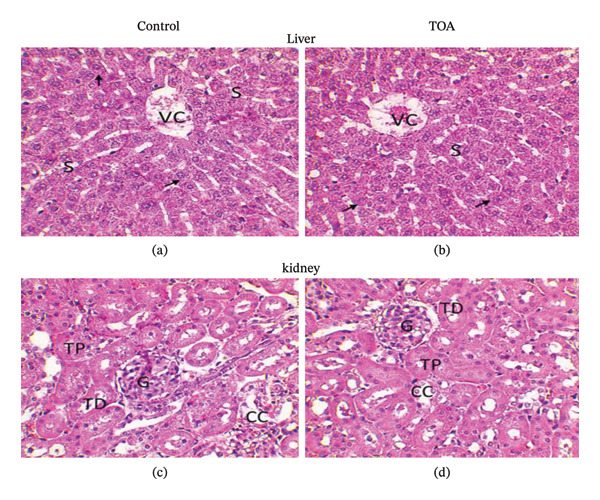
Hepatic (a, b) and renal (c, d) histological sections from rats treated with *Caesalpinia bonduc* extract following acute oral administration (2000 mg/kg). Liver sections show normal hepatocytes arranged in radial cords around the central vein with clearly visible sinusoids, while kidney sections display preserved glomeruli and renal tubules. Hematoxylin and eosin staining; original magnification × 400.

**FIGURE 2:**
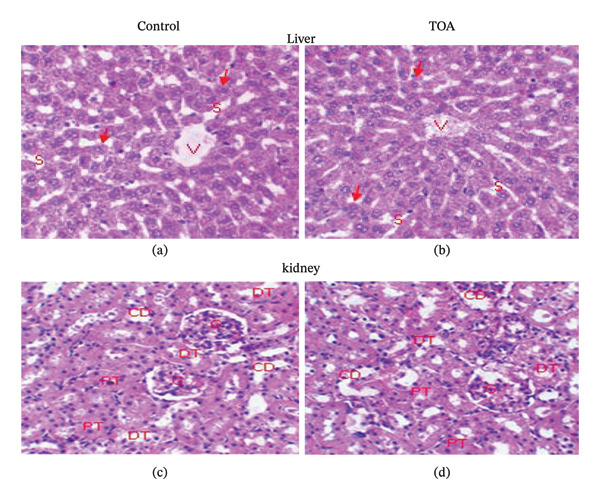
Hepatic (a, b) and renal (c, d) histological sections from rats treated with *Heliotropium indicum* extract following acute oral administration (2000 mg/kg). Normal hepatic and renal architectures are observed, comparable to control animals, with no histopathological lesions detected. Hematoxylin and eosin staining; original magnification × 400.

Histopathological examination of liver and kidney sections from rats treated with *Caesalpinia bonduc* and *Heliotropium indicum* extracts revealed no treatment‐related morphological alterations when compared with control animals (Figures 1 and 2).

In the acute oral toxicity study, liver sections from extract‐treated rats showed preserved hepatic architecture, with hepatocytes appearing normal and arranged in well‐defined radial cords surrounding the central vein (VC). Hepatic sinusoids (S) were clearly visible and regular, with no evidence of hepatocellular degeneration, necrosis, inflammatory infiltration, or vascular congestion (Figures 1(a), 1(b), 2(a), and 2(b)).

Similarly, kidney sections from treated animals exhibited normal renal histoarchitecture, characterized by intact glomeruli (G), well‐defined proximal and distal tubules (T), and normal collecting ducts (CC). No signs of tubular degeneration, glomerular damage, interstitial inflammation, or hemorrhage were observed in comparison with control rats (Figures 1(c), 1(d), 2(c), and 2(d)).

These findings indicate the absence of histopathological toxicity in the liver and kidneys following acute oral administration of the plant extracts at a dose of 2000 mg/kg.

## 4. Discussion

Medicinal plants play a crucial role in global health systems and represent a valuable source of bioactive compounds with diverse pharmacological properties. Understanding the phytochemical composition of these plants and evaluating their biological activities are essential steps toward validating their traditional uses and ensuring their safe therapeutic application [1].

The qualitative phytochemical screening conducted in this study revealed the presence of several classes of secondary metabolites, including tannins, alkaloids, leucoanthocyanins, reducing compounds, coumarins, quinone derivatives, and flavonoids. These findings are generally consistent with previous research describing the phytochemical composition of *Caesalpinia bonduc* and *Heliotropium indicum* [34, 35]. However, slight variations were observed, such as the detection of mucilage in the aerial parts of *H. indicum* but not in the roots of *C. bonduc*. Such differences may be attributed to variations in plant parts analyzed, environmental conditions, geographic origin, or extraction procedures.

Beyond their simple detection, these secondary metabolites are products of specific plant biosynthetic pathways (shikimate, acetate–malonate, and mevalonate pathways) and play essential ecological roles, including defense against microbial pathogens, herbivores, and environmental stress [36, 37]. For instance, phenolic compounds such as flavonoids and tannins are synthesized as part of plant defense systems and contribute to protection against oxidative stress and microbial invasion. This ecological function provides a biological rationale for their pharmacological activities in humans [38].

Although cyanogenic derivatives, anthracene, O‐heterosides, and C‐heterosides, were not detected in the analyzed plant materials, this observation should not be interpreted as definitive evidence of safety. Qualitative phytochemical screening remains preliminary and does not capture dose‐dependent toxicity, minor toxic constituents, or environmental contaminants. Therefore, safety conclusions must be supported by toxicological evaluation, as performed in the present study.

A limitation of the present study is the absence of quantitative phytochemical characterization using advanced analytical techniques such as HPLC–MS. Such analyses would allow precise identification of the bioactive compounds and support structure–activity relationship analysis.

Secondary metabolites identified in this study are known to exert a wide range of biological effects through well‐characterized mechanisms. Flavonoids and tannins can disrupt bacterial membranes, increase permeability, and induce leakage of intracellular components, while also inhibiting enzymes involved in nucleic acid synthesis [39, 40]. Alkaloids may intercalate into DNA and interfere with replication and transcription processes [41]. In addition, several phytochemicals have been shown to interfere with quorum‐sensing systems, thereby disrupting bacterial communication and virulence regulation [42]. These properties are consistent with their reported antibacterial, analgesic, anti‐inflammatory, and antioxidant activities [43, 44].

In the antibacterial assay, only the hydroethanolic extract of *C. bonduc* roots showed activity against uropathogenic strains, consistent with previous reports [35, 45, 46]. The moderate activity observed (high MIC values) likely reflects low concentrations of active compounds or antagonistic interactions within the crude extract. The bactericidal effect (MBC/MIC ≤ 4) suggests irreversible cellular damage, possibly through membrane disruption and protein denaturation induced by phenolic compounds [39].

The absence of detectable antibacterial activity in *H. indicum* extracts contrasts with some reports [34, 40] and may be explained by differences in extraction solvent, plant part, or experimental conditions. In particular, prolonged aqueous maceration may lead to degradation of thermolabile compounds or microbial contamination, affecting activity outcomes.

In contrast, neither the aqueous nor the hydroethanolic extracts of *Heliotropium indicum* exhibited detectable antibacterial activity in this study. This observation differs from some previously reported antimicrobial activities of this species [34, 47]. Such discrepancy may be attributed to differences in experimental conditions, extraction solvent, plant part used, bacterial strains tested, geographic origin of the plant material, and assay sensitivity. Furthermore, the preparation of aqueous extracts through prolonged maceration may lead to degradation of unstable phytochemicals or microbial contamination, which could affect biological activity measurements. These methodological limitations should therefore be considered when interpreting the results obtained with aqueous extracts.

Despite this lack of antibacterial activity, both plant species demonstrated the ability to inhibit biofilm formation. This apparent discrepancy is mechanistically justified, as antibiofilm activity does not necessarily require direct inhibition of planktonic bacterial growth. Indeed, natural compounds can disrupt quorum‐sensing pathways, inhibit bacterial adhesion, or interfere with extracellular polymeric substance synthesis without exerting bactericidal effects [26, 27, 48]. Thus, the inclusion of *H. indicum* extracts in antibiofilm assays is justified and highlights their potential as antivirulence agents rather than direct antimicrobials. The observed antibiofilm activity may be attributed to flavonoids, tannins, and saponins, which are known to interfere with early biofilm development and quorum‐sensing signaling [42, 49, 50].

Both plants also demonstrated significant analgesic activity in the acetic acid–induced writhing test. This effect is likely related to the inhibition of peripheral inflammatory mediators, particularly prostaglandins, through modulation of cyclooxygenase pathways by flavonoids and other phenolic compounds [51, 52]. These findings are consistent with earlier pharmacological studies reporting analgesic properties for *H. indicum* [53] and *C. bonduc* [46].

The diuretic activity observed may be explained by the modulation of renal function, particularly through increased glomerular filtration and reduced tubular reabsorption. This is consistent with the increased urinary excretion of Na^+^, K^+^, and Cl^−^ observed in this study [54].

Importantly, the antibacterial, antibiofilm, analgesic, and diuretic activities identified in this study can be mechanistically linked through the multifunctional nature of plant secondary metabolites. These compounds often exhibit pleiotropic effects, including membrane interaction, enzyme inhibition, and modulation of signaling pathways, resulting in a broad spectrum of pharmacological activities [39, 42].

The acute toxicity study demonstrated no mortality or significant physiological alterations, supporting a favorable preliminary safety profile. These findings are consistent with previous studies reporting low acute toxicity in the absence of significant hematological, biochemical, or histopathological changes [55, 56]. However, evidence of potential toxicity under chronic exposure conditions, particularly for *H. indicum*, highlights the need for long‐term safety evaluations [57].

Overall, the findings provide a coherent pharmacological rationale supporting the traditional use of *C. bonduc* and *H. indicum*, particularly in the management of infections, pain, and fluid retention. The observed activities align with their ethnomedicinal applications, where these plants are commonly prepared as decoctions or infusions for treating inflammatory and urinary disorders. Nevertheless, further studies are required to isolate active compounds, elucidate their mechanisms of action, and confirm their efficacy and safety in more advanced models.

## 5. Conclusion

This study provides preliminary experimental support for the ethnomedicinal use of *Caesalpinia bonduc* and *Heliotropium indicum* in the management of infectious, pain‐related, and fluid retention–associated conditions. Rather than demonstrating broad‐spectrum antibacterial efficacy, the findings suggest that the hydroethanolic extract of *C. bonduc* roots may contribute to antibacterial effects of moderate intensity, while both species exhibit consistent antibiofilm activity, which is particularly relevant in the context of persistent infections. The observed analgesic and diuretic effects further align with traditional therapeutic indications, supporting the rationale for their continued use in local healthcare practices. Acute toxicity assessment indicated no evidence of short‐term toxicity at the tested dose, suggesting a favorable preliminary safety profile under the experimental conditions, without implying long‐term safety.

Overall, these findings contribute to bridging traditional knowledge and experimental validation but remain exploratory in nature. Further investigations, including quantitative phytochemical characterization, bioactivity‐guided fractionation, and mechanistic studies, are required to identify the active constituents and to better define the pharmacological relevance and safety of these medicinal plants.

## Funding

No funding was received for this manuscript.

## Disclosure

The authors declare that no external individuals or third‐party services contributed to this work beyond the listed authors.

## Conflicts of Interest

The authors declare no conflicts of interest.

## Data Availability

The data that support the findings of this study are available from the corresponding author upon reasonable request.
